# Dissemination of *Fusarium proliferatum* by mealworm beetle *Tenebrio molitor*

**DOI:** 10.1371/journal.pone.0204602

**Published:** 2018-09-27

**Authors:** Zhiqing Guo, Katharina Pfohl, Petr Karlovsky, Heinz-Wilhelm Dehne, Boran Altincicek

**Affiliations:** 1 Laboratory of Quality & Safety Risk Assessment for Tobacco, Tobacco Research Institute of Chinese Academy of Agricultural Sciences, Qingdao, China; 2 Institute of Crop Science and Resource Conservation (INRES-Phytomedicine), Rheinische Friedrich-Wilhelms-University of Bonn, Bonn, Germany; 3 Molecular Phytopathology and Mycotoxin Research, University of Goettingen, Goettingen, Germany; AgroParisTech, FRANCE

## Abstract

**Background:**

Plant pathogenic fungi of the genus *Fusarium* infect a wide array of crops and produce numerous health-threatening mycotoxins. Recently, we found that larvae of the common pest of stored products *Tenebrio molitor* preferably fed on grains colonized with *Fusarium proliferatum*. We draw the hypothesis that the increased attractiveness of infected grains for mealworms facilitates dispersal of the fungus. In this work we examined the dissemination of *F*. *proliferatum* and further *Fusarium* spp. by adults of *T*. *molitor*.

**Results:**

Mealworm beetle *Tenebrio molitor* transmitted *Fusarium* species *F*. *avenaceum*, *F*. *culmorum*, *F*. *poae*, and *F*. *proliferatum* to wheat grains with varying efficiency. *F*. *proliferatum* was disseminated most efficiently: 20 days after feeding on *Fusarium* cultures, the beetles still transmitted *F*. *proliferatum* to most grains exposed to feeding. The transmission of *F*. *culmorum* gradually declined over time and the transmission of the other *Fusarium* spp. ceased completely 20 d after beetles feeding of fungal cultures. Propagules of *F*. *proliferatum* and *F*. *culmorum* were traceable in beetles' feces for 20 days while no colonies of *F*. *poae* and *F*. *avenaceum* were detectable after 5 days. Because *F*. *proliferatum* was transmitted by mealworms most efficiently, this species was further investigated. Mealworm beetles *T*. *molitor* preferred feeding on grains colonized with *F*. *proliferatum* as compared to uninfected grains. Male beetles infected with *F*. *proliferatum* transmitted the fungus by copulation.

**Conclusions:**

Efficient dissemination of *F*. *proliferatum* by mealworm beetle together with the feeding preference of the beetle for grains colonized with *F*. *proliferatum* show that the chemical phenotype of the fungus responsible for the enhanced attractiveness of infected grains is subjected to positive selection. This indicates that adaptation of *F*. *proliferatum* to transmission by insects involved an alteration of insects' feeding preferences.

## Introduction

Colonization of grains by *Fusarium* species impairs food security by diminishing the food quality by mycotoxins contamination. *F*. *proliferatum* has a wide host range. The pathogen has been most frequently isolated from maize, rice, sorghum and asparagus, but it was also found in banana [1], citrus fruits [2], date palm [3] and pine seedlings [4]. Since 1990, *F*. *proliferatum* is known to infect wheat [5–9]. *F*. *verticillioides* and *F*. *proliferatum* are the main source of mycotoxins fumonisins in food and feed products [8, 10]. Contamination with fumonisins raises food safety concerns because fumonisins exert acute toxicity as well as carcinogenic and teratogenic effects [11]. Many surveys reported association between high levels of fumonisins in maize with outbreaks of equine leukoencephalomalacia [12] and swine pulmonary edema [13]. Besides fumonisins, *F*. *proliferatum* is capable of synthesizing mycotoxins beauvericin [14], fusaproliferin [15], fusarins [16], and moniliformin [17].

Insects represent an important route in the transmission of numerous pathogens among plants [18]. Fermaud and Menn [19] reported that the grape berry moth *Lobesia botrana* transmitted fungus *Botrytis cinerea* from infected to healthy berries. Paine et al. [20] discussed that fungi of genus *Ophiostoma* may benefit from the association with bark beetles by being transported to new host trees. Beetles (*Dendroctonus* sp.) may benefit from the association by feeding on the fungus, or because pathogenic fungi kill the host tree. Adult corn earworm feeding on the honeydew secreted by *Claviceps africana* transmits ergot fungus from diseased to healthy panicles [21]. The rust fungus *Puccinia monoica* inhibits flowering of its host plants (*Arabis* species) and transforms infected leaves in a way mimicking true flowers, attracting pollinating insects [22]. Weevil benefit from feeding on rust-infected tissue and the fungus benefits from the transmission of its spores by weevils [23].

Mealworm beetle (syn. darkling beetle) *Tenebrio molitor* and its larvae (mealworms or yellow mealworms) are pests of stored grains. Recently we found that colonization of wheat grains with *Fusarium* spp. affected the food choice of mealworms [24]. Grains colonized with *F*. *proliferatum* were more attractive to larvae of *T*. *molitor* than uninfected grains. Because wheat plants can be systemically infected with *F*. *proliferatum* via colonized grains [5], we hypothesized in the current study that the attraction of *T*. *molitor* to grains infected with *F*. *proliferatum* facilitates dissemination of the fungus. Here we tested this hypothesis by investigating the dissemination of *F*. *proliferatum* and other *Fusarium* species in wheat grains by the beetles *T*. *molitor*.

## Materials and methods

### Organisms and media

*T*. *molitor* was reared on whole wheat flour with 5% yeast extract in a climate chamber in darkness at 27 ± 2°C and a relative humidity of 65 ± 5%. Before the experiments, the beetles were starved for 24 h for cleaning up the remaining food and also to ensure feeding on the mycelia [25]. For surface sterilization, beetles were carefully washed with autoclaved distilled water, soaked in 1.2–1.3% sodium hypochlorite for 3 min followed by 3-times rinsing with autoclaved distilled water for 2 min each. The beetles were dried on autoclaved paper and covered by sterile plastic lid under laminar flow cabinet.

Fungal strains (Table 1) have been described in previous work [24]. Fungal cultures were grown on potato dextrose agar (PDA, Merck, Darmstadt, Germany) plates in darkness at 23°C.

**Table 1:** Fungal strains.

Species	Host	Year	Origin
*Fusarium avenaceum* 1.27	wheat grains	2008	Poppelsdorf, Bonn, Germany
*Fusarium culmorum* 3.37	wheat	2004	Klein-Altendorf, Bonn, Germany
*Fusarium poae* DSM 62376	oat	1990	DSMZ, Braunschweig, Germany
*Fusarium proliferatum* 21.1	maize	2007	Hainichen, Germany
*Beauveria bassiana* Bea2	black vine weevil	1989	Stuttgart, Germany

CLA (Carnation Leaf-piece Agar) was prepared by placing two sterile pieces of carnation leaves onto 2% water agar plate (20 g agar in 1 L of water) [26]. Carnation leaves were harvested from plants grown without fungicide or insecticide application and dried in an oven at 70°C for 3–4 h until brittle. Dried leaves were sterilized with 1.5% sodium hypochlorite and dried at room temperature under a clean bench. PDB (potato dextrose broth) was purchased from Merck (Darmstadt, Germany). CZID (Czapek-Dox-Iprodione-Dichloran) agar was prepared as described [27, 28] and amended with streptomycin, ampicillin and chlortetracycline (Applichem, Darmstadt, Germany), each at 0.1 g/L.

### Fungal DNA content and survival of *F*. *proliferatum* in *T*. *molitor*

For the estimation of fungal DNA and mycotoxin content in the insects, ten *T*. *molitor* beetles for each of four time points were allowed to graze for 24 h on *F*. *avenaceum*, *F*. *culmorum*, *F*. *poae*, and *F*. *proliferatum* cultures on PDA plates and subsequently transferred into sterile Petri dishes with autoclaved wheat grains. Entomopathogenic fungus *B*. *bassiana* was used as a control. Beetles were harvested on 1, 5, 10 and 15 d and DNA content of *Fusarium* DNA and mycotoxins was determined as described below. To determine whether *F*. *proliferatum* survived in the bodies of beetles, 10 *T*. *molitor* beetles were fed *F*. *proliferatum* mycelia on PDA plates until death (longest time period 15 d). Dead beetles were frozen at -20°C, surface-sterilized with 1% sodium hypochlorite for 3 min, rinsed 3 times with sterile distilled water and placed on CZID plates. The outgrowing mycelia were monitored and examined for taxonomic affiliation with *F*. *proliferatum*.

### Dissemination of *Fusarium* spp. by *T*. *molitor*

Transmission of fungal inoculum by *T*. *molitor* to healthy grains was investigated by placing individual beetles that had been previously fed on fungal cultures into arenas with uncontaminated wheat grains and monitoring grain infection as well as the presence of fungal propagules in beetles' excreta over a time course. The experiment was started by feeding 10 mealworm beetles on cultures of *F*. *avenaceum*, *F*. *culmorum*, *F*. *poae*, *F*. *proliferatum*, and *B*. *bassiana* on PDA plates for 24 h. After feeding, the beetles were placed into sterile Petri dishes with 50–60 autoclaved wheat grains each, a single beetle per plate. The grains were changed every 24 h. After 1, 5, 10, 15, and 20 d, fifteen grains were randomly selected from each Petri dish, placed on CZID plates and kept in darkness at 25°C to determine fungal colonization. Grains with outgrowing mycelia were counted, generating a colonization score from 0 to 15 for each animal and time point. At the same time, excreta (feces) of the beetles were collected, mashed in sterile water and spread on CZID plates. Fungal colonies on the plates with excreta were counted, resulting in a cfu value for each animal and time point. The experiment was started with 10 beetles for each fungal species but not all beetles survived till the last sampling. *B*. *bassiana* proved lethal: all beetles died within 5 days after feeding. These data were excluded from the analysis. Some beetles fed on cultures of *Fusarium* spp. died, too, but at least 5 beetles from each *Fusarium* culture survived till the end of the experiment. Therefore we used data from 5 beetles for each *Fusarium* spp. in the analysis, keeping the number of replicates at the same value of 5. The entire experiment was repeated twice; because the results were essentially the same, only data from one experiment are shown.

### Food choice experiment with *F*. *proliferatum*-infected grains

Wheat grains were soaked in distilled water for 18 h at room temperature. 20 g of soaked grains with 5 ml water were autoclaved in 100 ml Erlenmeyer flask for 20 min at 121°C, inoculated with 2 ml *F*. *proliferatum* conidia (1.3×10^6^ spores/ml) and incubated for 7 days to ensure complete colonization. Control wheat grains were treated in a same way without inoculation. Food preference of mealworm beetle *T*. *molitor* was investigated in Petri dishes of a diameter 142 mm marked to generate four equal sectors in the form of pie slices. 6 g of non-colonized wheat grains each were placed into opposing sectors of the plates and 6 g grains colonized with *F*. *proliferatum* to the other sectors. Ten *T*. *molitor* beetles were placed into the center of the dishes. After 20 min in dark, beetles in each sector were counted. Controls were prepared in the same way with all four sectors filled with autoclaved not infected grains and two opposite sectors randomly selected for counting. Ten replicates with 10 animals each were performed and the experiment was repeated twice.

### Microscopic analyses

Beetles fed on 2-weeks-old *F*. *proliferatum* culture for 24 h were sputter-coated with gold and examined by scanning electronic microscopy on Phenom G2 Pro (Phenom World, Eindhoven, Netherland) for fungal mycelia and conidia attached to their body. Beetles tested for fungal colonization were surface sterilized with 1.2–1.3% sodium hypochlorite for 3 min, rinsed 3 times with sterile distilled water for 2 min and placed on CZID plates. Images of the specimens observed under light microscope were recorded with a camera incorporated to the Leitz DMRB Leica light microscope using software Diskus 4.2 (Hilgers, Königswinter, Germany).

### Re-isolation of fungi from grains and beetles

Contaminated wheat grains and excreta, gut and eggs of beetles were surface sterilized with 1.2–1.3% sodium hypochlorite and cultured on CZID plates. Single colony or hyphal tips were transferred to both PDA and CLA plates. The taxonomic affiliation of fungal isolates was confirmed by morphological features, such as pigmentation of the mycelia and the form of micro- and macro-conidia. For DNA extraction, single colonies were cultivated in 50 ml PDB at 25°C for 4 d. The mycelia were freeze-dried and ground into fine powder for DNA extraction.

### Mycotoxin analysis

Beetles were dried in vacuum at 40°C overnight and ground. Mycotoxins were extracted as described before [29]. Beauvericin, fumonisin B_1_ and enniatins were separated on an RP column at 40°C followed by electrospray ionization in a positive mode and analysis on ion trap 500 MS (Varian, Darmstadt, Germany) [29]. The samples for trichothecenes B and zearalenone quantification were defatted with cyclohexane. Deoxynivalenol and zearalenone were separated on the same HPLC system and detected by tandem mass spectrometry using triple quadrupole 1200 L (Varian, Darmstadt, Germany) according to published methods [30, 31]. Two mass transitions were used for each toxin.

Calibration curves were constructed using analytical standards dissolved in methanol/water (1:1). The estimated limits of quantification (LOQ) and detection (LOD) for beauvericin and enniatins A, B, A_1_, and B_1_ were 150 ng/g and 60 ng/g, respectively. LOQ and LOD for fumonisin B_1_ were 390 ng/g and 190 ng/g, respectively. LOD for deoxynivalenol and zearalenone were 200 ng/g and 20 ng/g, respectively.

### DNA extraction, species-specific detection and quantification of fungal DNA

Total DNA of *T*. *molitor* was extracted using a CTAB method [32] and dissolved in TE buffer (10 mM Tris, 1 mM EDTA, pH 8.0). The quality and quantity of DNA were estimated by gel electrophoresis in 0.8% agarose gels (Cambrex, Rockland, USA) prepared in TAE buffer [33]. The electrophoresis was carried out at 4 V/cm for 60 min. The gel was stained with ethidium bromide (2 mg/l) and documented with a digital imaging system (Vilber Lourmat, Marne la Vallee, France). Prior PCR analysis, an inhibition assay was carried out to test the effect of matrix on DNA amplification.

DNA of fungal cultures re-isolated from wheat grains, insect excreta, and gut and eggs and grown in PDB was extracted using DNeasy Plant Mini kit (QIAGEN, Hilden, Germany) according to the manufacturer’s protocol. The DNA quality was monitored by electrophoresis in 1.0% agarose gels (Agarose NEEO Ultra-Quality, Carl Roth, Karlsruhe, Germany) prepared in TAE buffer [33]. The electrophoresis was carried out at 4 V/cm for 30 min. The agarose gel was stained with 10,000 × dilution Gel Red (Biotium, Darmstadt, Germany) and documented with a digital imaging system (Gel Doc, Bio-Rad, Munich, Germany).

Thermocycler (CFX384, BioRad, Munich, Germany) was used for real-time PCR analysis. Primers MGBF/R [34] and Fp 82F/R [35] were used for species-specific quantification of *F*. *avenaceum* and *F*. *poae*, respectively. The reactions were carried out in polymerase buffer (16 mM (NH_4_)_2_SO_4_, 67 mM Tris-HCl, 0.01% (v/v) Tween-20, pH 8.8 at 25°C) with 0.15 mM of each dNTP (Bioline, Luckenwalde, Germany), 2.5 mM MgCl_2_, 0.1 U of Taq DNA polymerase (BIOTaq, Bioline, Luckenwalde, Germany), 0.3 μM of each primer, and 0.1 × SYBR Green I (Invitrogen, Karlsruhe, Germany). *F*. *culmorum* and *F*. *proliferatum* DNA was quantified according to established protocols [29, 32]. The lowest standards set as limits of quantification were 0.169 pg/μl for *F*. *avenaceum*, *F*. *culmorum*, *F*. *poae* and 2.09 fg/μl for *F*. *proliferatum*.

Taxonomic identity of re-isolated *F*. *proliferatum* was confirmed by PCR with species-specific primers PRO1/PRO2 [36]. The reaction mixture contained Dream Taq buffer (Thermo Fisher Scientific, Darmstadt, Germany) with 4 mM MgCl_2_, 0.2 mM of each of the four deoxynucleoside triphosphates, 0.1 U of Dream Taq DNA polymerase (Thermo Fisher Scientific, Darmstadt, Germany), 0.5 μM of each primer and 2 μl sample DNA. The following cycling conditions were used: 1 cycle of 10 min at 94°C, 35 cycles of 60 s at 94°C, 30 s at 60°C, and 60 s at 72°C, followed by a final extension cycle at 72°C for 10 min [37]. 1% agarose gel was prepared in TAE buffer [33] and the electrophoresis was carried out at 4 V/cm for 30 min. The gel was stained with Gel Red and documented with a digital imaging system (Bio-Rad, Munich, Germany).

### Statistical analyses

Statistical analysis was performed with R 3.5.0 [38]. Data for infected grains were analyzed using logistic regression. Colonies counts were analyzed using a log-linear model. The results of the food choice experiments were evaluated using Chi-Square Goodness of Fit test. Box plots drawn using Excel 2016 show interquartile range with the median and mean; whiskers indicate the largest and smallest observation or 1.5-fold of the interquartile range, whichever was smaller/larger. The number of replicates and the number of grains or animals per replicate are specified in the description of the experiments in Material and Methods section.

## Results

### Dissemination of *Fusarium* spp. to new grains by mealworm beetle *T*. *molitor*

**Fig 1:**
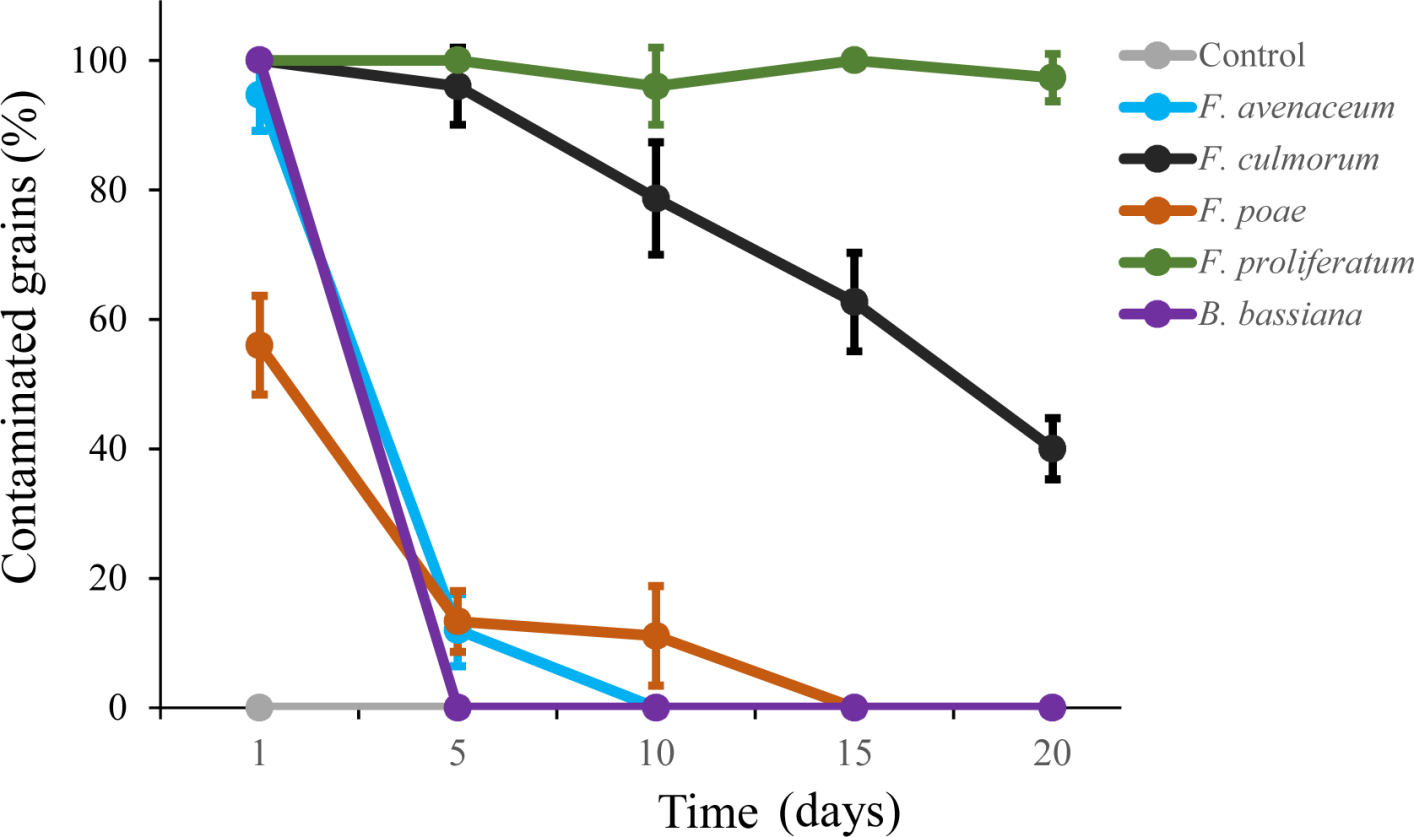
Transmission of *Fusarium* spp. to wheat grains by *Tenebrio molitor*. The graph shows percentage of wheat grains that became infected with *Fusarium* spp. after exposure to beetles of *T*. *molitor* that were previously fed on fungal cultures. Means ± S.D. are shown (n = 10).

Transmission of fungal inoculum to healthy grains by the beetle *T*. *molitor* was investigated by placing beetles fed on fungal cultures into Petri dishes with wheat grains. The grains were replaced daily and their contamination with *Fusarium* spp. was monitored for 20 d (Fig 1). *F*. *proliferatum* was disseminated most efficiently: no decline in the contamination of new grains with *F*. *proliferatum* was observed with contamination rates remaining above 90% till the end of the experiment. The contamination with *F*. *culmorum* gradually decreased over time while *F*. *avenaceum* and *F*. *poae* became undetectable after 10 d and 15 d, respectively. *B*. *bassiana* killed all beetles within 5 d, preventing transmission to new grains. The data fitted a generalized linear model with quasibinominal error structure and revealed that the contamination rates of *F*. *proliferatum* were significantly high (p < 0.01) when compared to that of *F*. *avenaceum*, *F*. *poae* and control. The original data were shown in S1 Table.

### Survival of propagules of *Fusarium* spp. in the digestive track of mealworm beetle *Tenebrio molitor*

**Fig 2:**
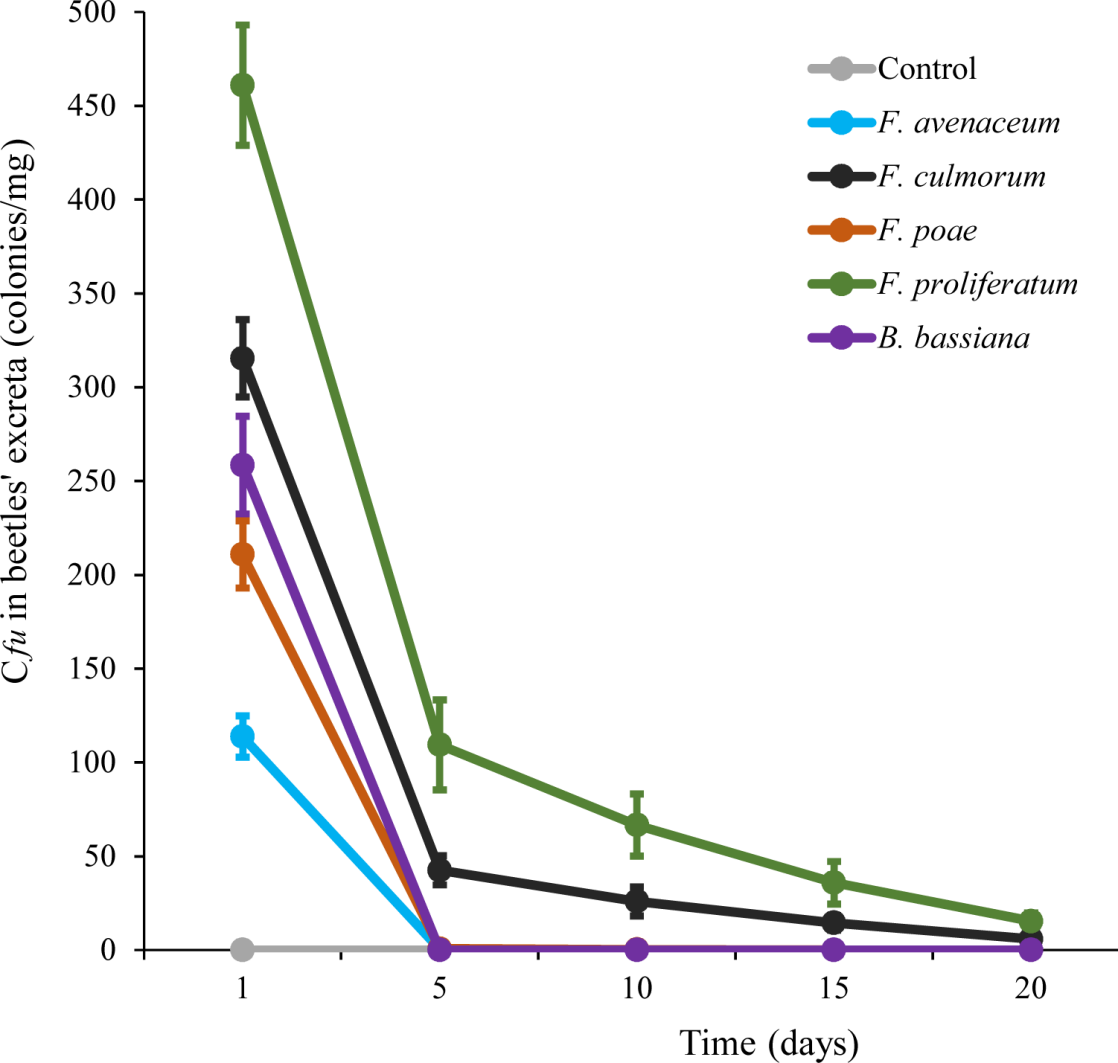
Dissemination of propagules of *Fusarium* species by feces of mealworm beetle *Tenebrio molitor* that has been fed on *Fusarium* cultures. The density of fungal propagules determined as the number of Cfu (colony forming units) in the excreta of *T*. *molitor* adults fed on cultures of *Fusarium* spp. is shown as mean ± S.D.

Dissemination of ingested fungal propagules by feces is only possible if the propagules survive the gut passage. To investigate this possibility, feces of beetles fed on fungal cultures were collected for 20 d, suspended in water and plated on *Fusarium*-specific agar media. Propagules of all fungal *Fusarium* species tested survived the gut passage (Fig 2). *F*. *proliferatum* was disseminated most efficiently, followed by *F*. *culmorum*. The density of *F*. *avenaceum* and *F*. *poae* propagules in the excreta declined rapidly, becoming undetectable after 5 d. The data fitted a generalized linear model and revealed that colony forming units of *F*. *proliferatum* were significantly different (p < 0.01) compared to that of *F*. *avenaceum*, *F*. *culmorum* and *F*. *poae*. Dissemination of *B*. *bassiana* could not be detected because all beetles fed on *B*. *bassiana* culture died within 5 d. No colonies of *Fusarium* spp. grew out of feces of beetles that were not fed on *Fusarium* cultures. The original data were shown in S2 Table.

### Fungal proliferation and mycotoxin accumulation in *T*. *molitor* beetles

Dissemination of fungal propagules by mealworm beetles is expected to last longer if the fungus multiplies in beetles' digestion track. *T*. *molitor* beetles were allowed to graze on *F*. *avenaceum*, *F*. *culmorum*, *F*. *poae*, *F*. *proliferatum*, and *B*. *bassiana* cultures, transferred into sterile Petri dishes with autoclaved wheat grains, harvested at different time points and the content of *Fusarium* DNA in the beetles was determined. DNA of all *Fusarium* species except *F*. *culmorum* was detected in most beetles until the last time point at 15 d (Table 2). Beetles fed on *F*. *culmorum* died within 10 d. Because only a single animal fed on *F*. *culmorum* survived till 15 d, the result of qPCR analysis was disregarded. Large variation in the content of fungal DNA among individuals at all time points (Table 2) prevented us from testing whether fungal biomass in the beetles during the study period was growing.

**Table 2:** Fungal DNA in beetles grazing on *Fusarium* spp. mycelia and conidia.

Fungal species	Species-specific fungal DNA in beetles (ng/g)			
	1 d	5 d	10 d	15 d
*Fusarium avenaceum*	87 ± 27 (7/10)	47 ± 18 (5/10)	106 ± 22 (9/10)	153 ± 67 (4/4)
*Fusarium culmorum*	110 ± 16 (10/10)	34 ± 8 (7/10)	186 ± 35 (9/10)	<LOQ (1/1)
*Fusarium poae*	3 (1/10)	5 ± 4 (2/10)	41 ± 40 (3/10)	3 ± 1 (7/8)
*Fusarium proliferatum*	258 ± 45 (10/10)	35 ± 4 (8/10)	100 ± 30 (10/10)	29 ± 14 (8/8)

Means and standard error of the mean of positive samples are shown. The number of beetles with detectable fungal DNA and the total number of beetles investigated are shown in brackets.

Mycotoxin content in beetles fed on *Fusarium* cultures was analyzed. Beauvericin was produced by three *Fusarium* species including the most efficiently disseminated *F*. *proliferatum*. Examination of the presence of beauvericin in the beetles at different time points showed that beetles fed on *F*. *poae* were consistently contaminated with beauvericin, while beetles grazing on *F*. *proliferatum* only rarely contained beauvericin (Table 3). Beetles consuming *B*. *bassiana* died within 5 days; all beetles contained beauvericin at this time point (Table 3). Deoxynivalenol, nivalenol and zearalenone were not detectable in beetles fed on *F*. *culmorum* (Table 4). Enniatins were detected in beetles fed on all fungal species; it turned out that wheat grains used for the experiment were naturally contaminated with enniatins at 9.0 ± 1.0 ng/g.

**Table 3:** Beauvericin contamination of *T*. *molitor* beetles.

Fungal species	Time	Beetles contaminated with beauvericin
*F*. *poae*	1 d	4/10^a^
	5 d	1/10
	10 d	10/10
	15 d	4/8
*F*. *proliferatum*	1 d	2/10
	5 d	2/10
	10 d	1/10
	15 d	0/9
*B*.*bassiana*	1 d	1/10
	5 d	10/10
	10 d	/
	15 d	/

The limits of quantification (LOQ) for beauvericin was 150 ng/g. Slash indicates that all beetles died before sampling.

^a^ Number of beetles contaminated with beauvericin/total number of beetles.

**Table 4:** Mycotoxin content in *T*. *molitor* beetles.

Fungal species		Beauvericin	Fumonisin B_1_	Enniatin A	Enniatin B	Enniatin A_1_	Enniatin B_1_	Deoxynivalenol	Zearalenone
(ng/g)									
**Control**	1 d	<LOQ	<LOQ	<LOQ	6 ± 1	<LOQ	2 ± 0	<LOQ	<LOQ
	5 d	<LOQ	<LOQ	<LOQ	5 ± 0	<LOQ	7 ± 4	<LOQ	<LOQ
	10 d	<LOQ	<LOQ	<LOQ	15 ± 1	2 ± 1	7 ± 1	<LOQ	<LOQ
	15 d	<LOQ	<LOQ	<LOQ	5± 0	<LOQ	3 ± 0	<LOQ	<LOQ
***F*. *avenaceum***	1 d	<LOQ	<LOQ	<LOQ	246 ± 32 ^b^	275 ± 8 ^b^	228 ± 42	/	/
	5 d	<LOQ	<LOQ	<LOQ	406 ± 47	261 ± 18 ^b^	296 ± 30	/	/
	10 d	<LOQ	<LOQ	<LOQ	552 ± 49 ^b^	291 ± 22 ^b^	355 ± 37 ^b^	/	/
	15 d	<LOQ	<LOQ	<LOQ	215 ± 20 ^b^	<LOQ	177 ± 5 ^b^	/	/
***F*. *poae***	1 d	251 ± 28 ^b^	<LOQ	<LOQ	<LOQ	202 ± 10 ^b^	156 ± 9	/	/
	5 d	176 ^b^	<LOQ	<LOQ	365 ^b^	259 ± 25	220 ± 32	/	/
	10 d	843 ± 187 ^b^	<LOQ	<LOQ	234 ± 12 ^b^	336 ± 26	305 ± 33	/	/
	15 d	215 ± 28 ^b^	<LOQ	212 ^b^	<LOQ	221 ± 7	175 ± 6	/	/
***F*. *proliferatum***	1 d	1383 ± 1171 ^b^	<LOQ	<LOQ	218 ± 70 ^b^	265 ± 74 ^b^	230 ± 55	/	/
	5 d	499 ± 330 ^b^	<LOQ	<LOQ	257 ± 17 ^b^	207 ± 8 ^b^	203 ± 15	/	/
	10 d	<LOQ	<LOQ	<LOQ	466 ± 46 ^b^	209 ± 14 ^b^	309 ± 31 ^b^	/	/
	15 d	<LOQ	<LOQ	<LOQ	185 ± 11 ^b^	225 ± 6 ^b^	166 ± 4	/	/
***F*. *culmorum***	1 d	/	/	/	/	/	/	<LOQ	<LOQ
	5 d	/	/	/	/	/	/	<LOQ	<LOQ
	10 d	/	/	/	/	/	/	<LOQ	<LOQ
	15 d	/	/	/	/	/	/	<LOQ	<LOQ
***B*. *bassiana***	1 d	161^b^	<LOQ	<LOQ	240 ± 15 ^b^	330 ± 33	283 ± 37 ^b^	/	/
	5 d	1759 ± 511	<LOQ	413 ± 81 ^b^	232 ± 26	279 ± 15	224 ± 14	/	/

Notes

The values indicated mean values and respective standard error of mean. Slash indicated that the mycotoxin was not analyzed. The limits of quantification (LOQ) of beauvericin, enniatin A, enniatin B, enniatin A1, enniatin B1 were 150 ng/g while LOQ of fumonisin B1 was 390 ng/g.

^b^ not all 10 samples contained detectable amount of the toxin.

### Food preference of *T*. *molitor* for grains colonized with *F*. *proliferatum*

**Fig 3:**
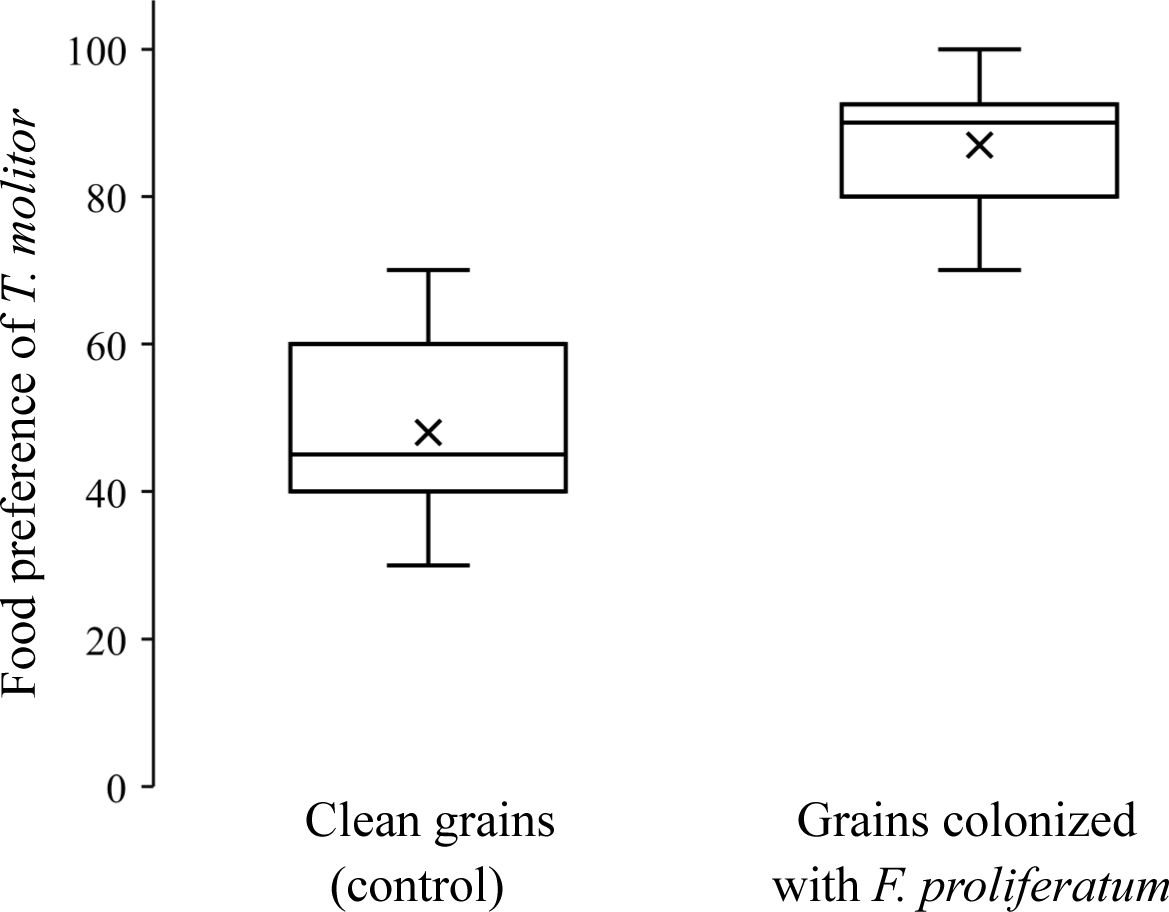
Food preference of *Tenebrio molitor* for wheat grains colonized with *Fusarium proliferatum*. Mealworm beetles *T*. *molitor* were offered wheat grains colonized with *F*. *proliferatum* and uninfected grains. After 20 min, beetles feeding on grains with *F*. *proliferatum* were counted and the count was expressed as a percentage of all beetles in the arena. In the control, both food choices consisted of uninfected grains. The preference of the beetles for infected grains was statistically significant (chi-square test, Χ^2^ = 59.3, p < 0.001).

Because of long-lasting dissemination of *F*. *proliferatum* by mealworm beetles that were previously fed on *F*. *proliferatum* (Fig 1), we investigated how colonization with *F*. *proliferatum* affected the food preference of the beetles. *T*. *molitor* strongly preferred grains infected with *F*. *proliferatum* over uninfected grains (Fig 3). The experiment was repeated twice with essentially the same result.

### *F*. *proliferatum* propagules adhere to the body of *T*. *molitor*

**Fig 4:**
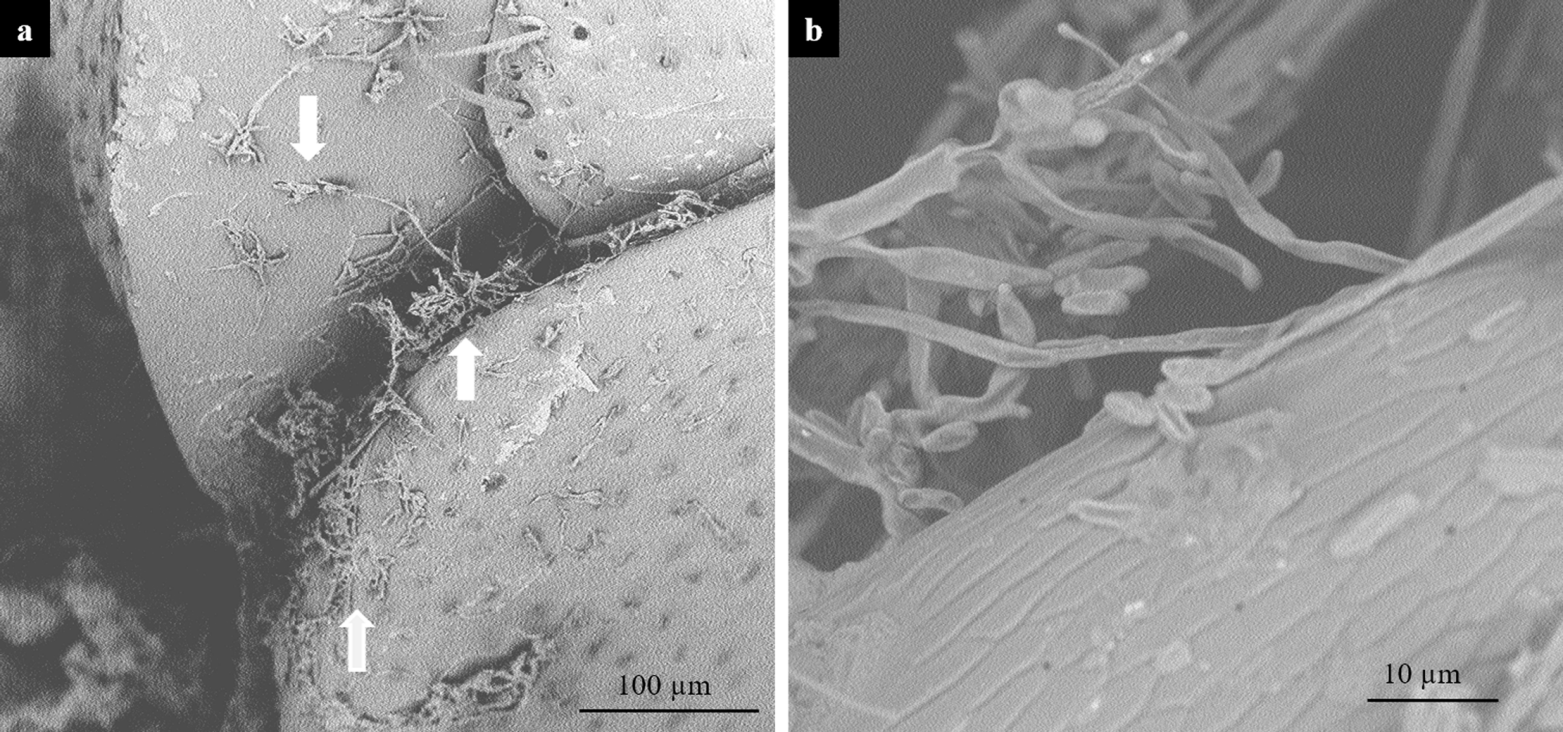
Hyphae and conidia on the body of *Tenebrio molitor* fed on *Fusarium proliferatum*. Scanning electron microscopy photographs show *F*. *proliferatum* hyphae (a) and conidia (b) adhering to the cuticle of *T*. *molitor* beetles that were fed on *F*. *proliferatum* culture.

**Fig 5:**
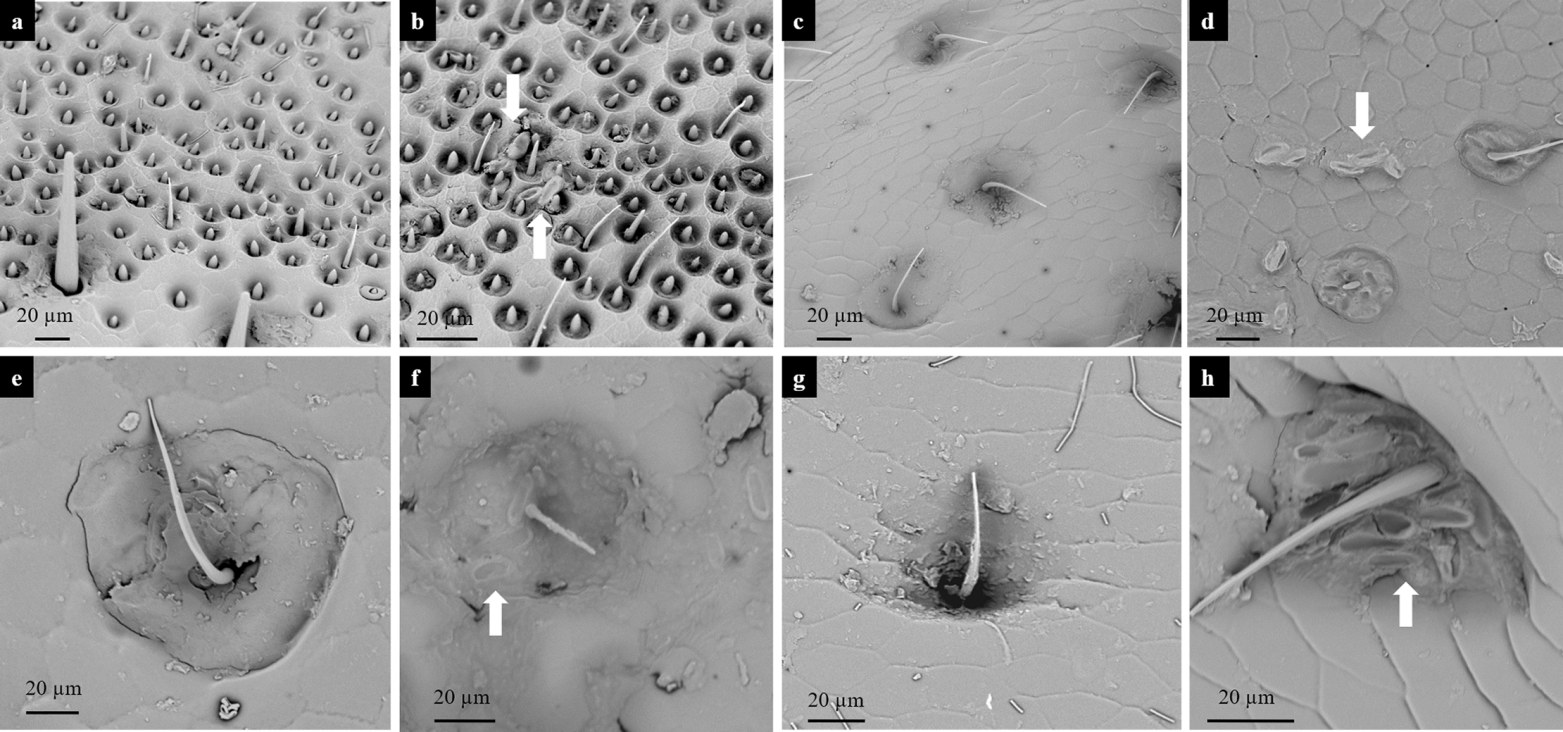
Hyphae and conidia on the extremities of *Tenebrio molitor* fed on *Fusarium proliferatum*. Scanning microscopy photographs show *F*. *proliferatum* conidia attached to the antennae (b; a is a control), mouthparts (d; c is a control), wings (f, e is a control), and legs (h; g is a control).

The presence of propagules of the *F*. *proliferatum* on the surface of beetles' bodies was examined by electron microscopy. Fungal mycelia (Fig 4A) and conidia (Fig 4B) were observed on the surface of beetles fed on *F*. *proliferatum* cultures. The fungus isolated from these samples was confirmed to be *F*. *proliferatum*. We also found fungal conidia attached on antennae (Fig 5B with the control 5A), mouthparts (Fig 5D with the control 5C), wings (Fig 5F with the control 5F) and legs (Fig 5H with the control 5G) of the beetles. No mycelia or conidia were found on beetles that did not have contact with a fungal culture.

**Fig 6:**
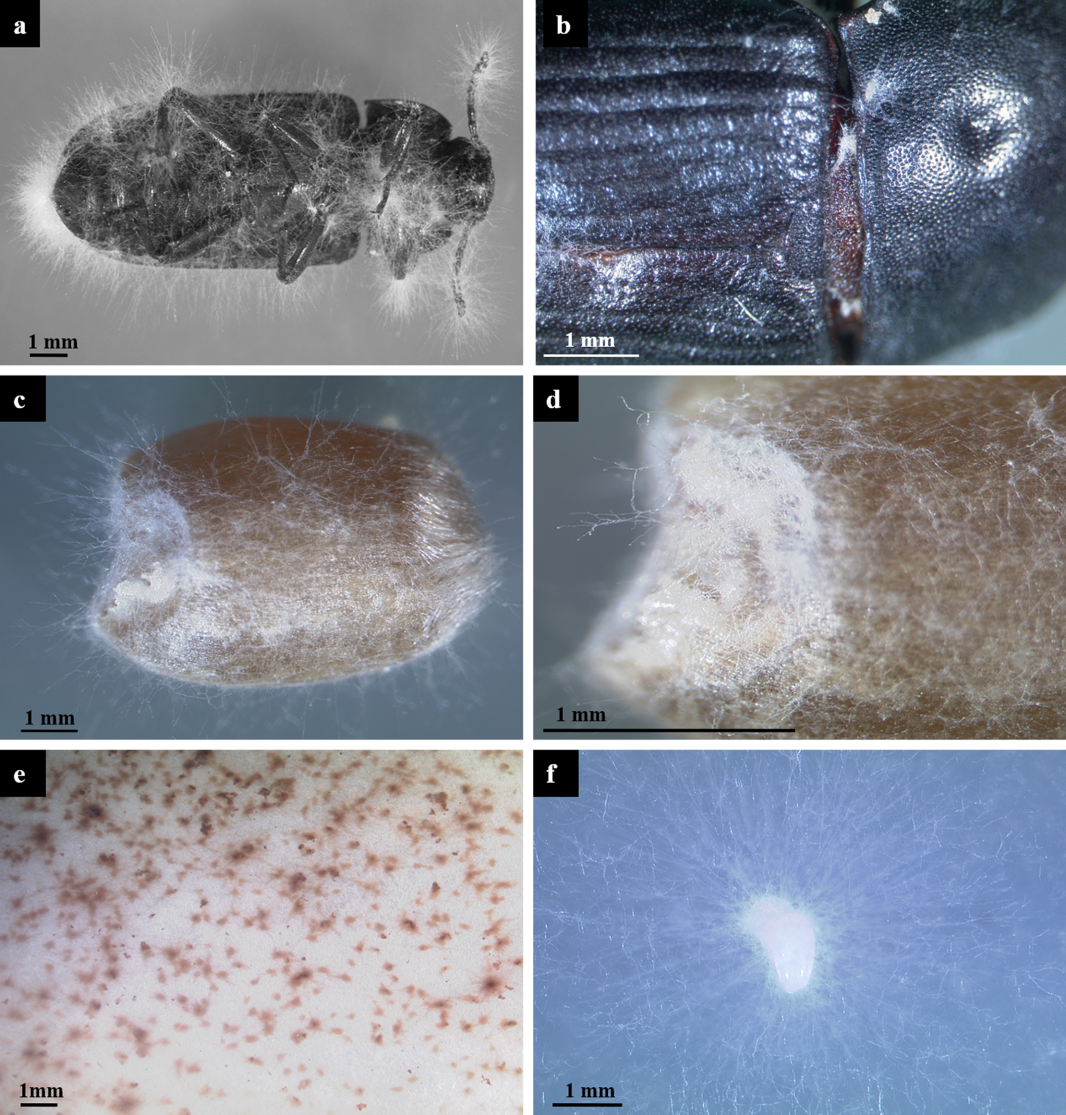
Growth of *Fusarium proliferatum* from dead bodies, feces and eggs of *Tenebrio molitor* and from contaminated grains. The upper part of the figure shows fungal hyphae growing out of the bodies of dead beetles fed on *F*. *proliferatum* (a and b) and wheat grains contaminated with *F*. *proliferatum* by the beetles (c and d). The lower part of the figure shows fungal colonies growing from feces of beetles suspended in water and plated on agar medium (e), and an egg of *T*. *molitor* contaminated with *F*. *proliferatum* via copulation (f).

Fungal mycelia grew out of dead bodies of beetles fed on *F*. *proliferatum* cultures (Fig 6A and 6B). The mycelia were confirmed to belong to *F*. *proliferatum* by PCR with species-specific primers.

### Transmission of *F*. *proliferatum* to the next generation of *T*. *molitor* by copulation

The long-lasting dissemination of *F*. *proliferatum* by mealworm beetles (Fig 4) raised the question whether the fungus can be transmitted from male beetles to offspring by copulation. Five male beetles fed on *F*. *proliferatum* for 48 h were mated with five females. After 7 d, the females were dissected and 9–11 eggs from each female (a total of 50 eggs) were picked up with sterilized forceps and placed on CZID plates for 3–4 d at 25°C. The emergence of *Fusarium* mycelia (Fig 6F) was recorded. 50 eggs from 5 female beetles after copulation with males fed on clean grains were used as negative controls. Out of the eggs from female beetles inseminated by males fed on *F*. *proliferatum*, outgrowth of fungal mycelia was detected in 30 eggs, with 44–80% contaminated eggs per beetle. The taxonomic assignment of fungal colonies to *F*. *proliferatum* was confirmed by morphology and species-specific PCR. Except for a single case of contamination with an *Aspergillus* species, no fungal growth was observed on control eggs.

## Discussion

Seed-borne infection of wheat plants with *F*. *proliferatum* causes systemic colonization of the plants and mycotoxins accumulation in wheat grains [5]. Transmission of *F*. *proliferatum* in storage from infected to uninfected grains might increase the incidence of wheat plants grown from stored seeds. This work provides evidence that *T*. *molitor* is capable of disseminating *F*. *proliferatum* efficiently among stored grains.

It is well known that pest insects may increase mold incidence in stored grains [39]. To our knowledge, this phenomenon has not been exploited in an ecological context, especially regarding the food preference of pests. Our previous work showed that colonization of grains with *F*. *proliferatum* increased their attractiveness to mealworms (larvae of *Tenebrio molitor*) [24]. In the current work, adult beetles of *T*. *molitor* exhibited a strong preference for grains colonized with *F*. *proliferatum* and *T*. *molitor* beetles fed on *F*. *proliferatum* disseminated the fungus to fresh grains at a high rate for an extended period of time. It is tempting to hypothesize that the increased attractiveness of grains infected with *F*. *proliferatum* enhances the fitness of the fungus by accelerating its dissemination. Positive selection may maintain the chemical phenotype of *F*. *proliferatum* that is responsible for its attractiveness to the beetles. Furthermore, the dissemination of *F*. *proliferatum* by *T*. *molitor* may select *F*. *proliferatum* strains that allow host beetles to survive the infection for an extended time period. A rigorous test of this hypothesis would require quantitative estimation of fungal fitness, accounting for dissemination by *T*. *molitor* as well as for the loss of fungal propagules due to feeding. The strong food preference of the beetles for infected grains and the high efficiency and long persistence of the dissemination of fungal propagules by *T*. *molitor* however suggest that the dissemination of *F*. *proliferatum* by insects is an integral part of its life history.

Why was only *F*. *proliferatum* disseminated efficiently by *T*. *molitor*, while the other *Fusarium* species disappeared completely 20 days after the contact with the beetles? All four *Fusarium* species infect flowers of grain crops, followed by the colonization of developing grains. In grain crops, *F*. *proliferatum* occurs mainly in maize; infection of wheat with *F*. *proliferatum* has recently been established but the fungus appears to be only a minor pathogen of wheat. It has not been reported from other small-grain cereals so far. The other three *Fusarium* species occur frequently on maize as well as on small-grain cereals and all of them are components of the *Fusarium* Head Blight disease complex. None of the species form races. Thus specialization for host plants is unlikely to explain the differences in the dissemination rates of *Fusarium* species by *T*. *molitor*.

*F*. *proliferatum* however differs from the other three *Fusarium* species regarding its association with insects. A recent survey of entomopathogenic *Fusarium* species associated with *Tribolium* beetles recovered only two *Fusarium* species: *F*. *keratoplasticum* and *F*. *proliferatum* [40]. Furthermore, *F*. *proliferatum* was used to control wheat flour beetle *Tribolium castaneum* [41]. The ability of *F*. *proliferatum* to infect insects may be related to its long-term dissemination by *T*. *molitor* beetles that had been exposed to *F*. *proliferatum* culture. Association of the other three *Fusarium* species used in this study with insects has been occasionally reported, too. For instance, *F*. *avenaceum*, *F*. *culmorum*, and *F*. *poae* were isolated from the housefly *Musca domestica*, the clover leaf weevil *Hypera punctata*, and the grass hopper *Melanoplus bivittatus*, respectively [42]. These reports however reflect rare and spurious observations rather than established fungus-insect associations. A recent review concluded that in spite of an increase of *Fusarium* diseases of grain crops with herbivore infestation reported in many studies, vector activity of the insects has rarely been demonstrated [43]. In a few publications reporting direct transmission of *Fusarium* spp. by insects, either the acquisition of inoculum from an ecologically relevant source was not shown or the success rate of the transmission was not determined [43].

Propagules of *F*. *proliferatum* and to a lesser extent other *Fusarium* species survived passage through the digestion tract of the insects. Survival of a gut passage is a key feature of entomopathogenic fungi [44]. In contrast to entomopathogenic fungus *B*. *bassiana*, *F*. *proliferatum* has not killed beetles that ingested fungal cultures. Low concentrations of beauvericin found in beetles fed on *F*. *proliferatum* indicate that the fungus ceased producing the mycotoxin in insect bodies, which may be part of its adaptation to dissemination by the beetles. Long survival of a plant pathogenic fungus in the digestion track of pests points out at a potentially important route of disease spread, establishing a new link between storage pest management and the control of fungal diseases.

Our results showed that *F*. *proliferatum* can be transmitted by mating of *T*. *molitor* to the eggs, which may lead to dissemination of the fungus by the new generation of the pest. Transmission of fungi to next generations by mating has been described for classical entomopathogens *Metarhizium anisopliae* and *Beauveria bassiana* [45]. Thus survival in the digestion track of insects and transmission to a next generation via copulation are features shared by *F*. *proliferatum* and entomopathogenic fungi. Apart from insect hosts to which it is pathogenic [41], *F*. *proliferatum* appears to be adapted to insects to which it does not cause visible disease. The adaptation of *F*. *proliferatum* to *T*. *molitor* may not allow appreciable proliferation of the fungus within insect host (Table 2) but it certainly facilitates its dissemination.

While preferentially feeding on grains infected with *Fusarium* spp., *T*. *molitor* is likely to ingest mycotoxins. Furthermore, fungus surviving in the digestive track of the insect might continue producing mycotoxins. Contamination of *T*. *molitor* with mycotoxins is relevant for food safety because the insect belongs to alternative proteins sources for food [46] and for life support systems for astronauts [47]. Surprisingly, mycotoxin fumonisin B_1_ was not detectable in beetles fed on *F*. *proliferatum*. The synthesis of fumonisin in *F*. *verticillioides*, which is closely related to *F*. *proliferatum*, underlies a complex control with epigenetic regulation [48]. We assume that the amount of fumonisin ingested with fungal mycelia was too small to be detected and that the synthesis of fumonisins was down-regulated in the insects. Alternatively, insects may have detoxified fumonisin, as indicated by the results of feeding and injecting mealworms with pure fumonisin B_1_ [49].

In conclusion, our study has shown that beetles of *T*. *molitor* are attracted to wheat grains infected with *F*. *proliferatum* and serve as a vehicle for the transmission of the fungus to uninfected grains. *F*. *proliferatum* survived passage via the digestive tract of insects and was transmitted to eggs by copulation, which suggests that *F*. *proliferatum* is adapted to dissemination by insect hosts.

## Supporting information

S1 Table Original data of cfu in beetles’ excreta. (ZIP)

S2 Table Original data of contaminated wheat grains. (ZIP)
